# Predicting fitness in *Mycobacterium tuberculosis* with transcriptional regulatory network-informed interpretable machine learning

**DOI:** 10.3389/ftubr.2025.1500899

**Published:** 2025-04-02

**Authors:** Ethan Bustad, Edson Petry, Oliver Gu, Braden T. Griebel, Tige R. Rustad, David R. Sherman, Jason H. Yang, Shuyi Ma

**Affiliations:** ^1^Center for Global Infectious Disease Research, Seattle Children's Research Institute, Seattle, WA, United States; ^2^Center for Emerging and Re-Emerging Pathogens, Rutgers New Jersey Medical School, Newark, NJ, United States; ^3^Department of Chemical Engineering, University of Washington, Seattle, WA, United States; ^4^Department of Microbiology, University of Washington, Seattle, WA, United States; ^5^Department of Microbiology, Biochemistry and Molecular Genetics, Rutgers New Jersey Medical School, Newark, NJ, United States; ^6^Department of Pediatrics, University of Washington, Seattle, WA, United States; ^7^Pathobiology Graduate Program, Department of Global Health, University of Washington, Seattle, WA, United States

**Keywords:** *Mycobacterium tuberculosis*, transcriptional regulation, network inference, network modeling, interpretable machine learning, growth regulation, stress adaptation, hypoxia

## Abstract

**Introduction:**

*Mycobacterium tuberculosis* (Mtb) is the causative agent of tuberculosis disease, the greatest source of global mortality by a bacterial pathogen. Mtb adapts and responds to diverse stresses, such as antibiotics, by inducing transcriptional stress response regulatory programs. Understanding how and when mycobacterial regulatory programs are activated could inform novel treatment strategies that hinder stress adaptation and potentiate the efficacy of new and existing drugs. Here, we sought to define and analyze Mtb regulatory programs that modulate bacterial fitness under stress.

**Methods:**

We assembled a large Mtb RNA expression compendium and applied this to infer a comprehensive Mtb transcriptional regulatory network and compute condition-specific transcription factor activity (TFA) profiles. Using transcriptomic and functional genomics data, we trained an interpretable machine learning model that predicts Mtb fitness from TFA profiles.

**Results:**

We demonstrated that a TFA-based model can predict Mtb growth arrest and growth resumption under hypoxia and reaeration using gene expression data alone. This model also directly elucidates the transcriptional programs driving these growth phenotypes.

**Discussion:**

These integrative network modeling and machine learning analyses enable the prediction of mycobacterial fitness across different environmental and genetic contexts with mechanistic detail. We envision these models can inform the future design of prognostic assays and therapeutic interventions that can cripple Mtb growth and survival to cure tuberculosis disease.

## 1 Introduction

*Mycobacterium tuberculosis* (Mtb) is a highly successful pathogen, infecting 10.6 million people and killing over 1 million people worldwide each year (1). A key factor for Mtb's success is its ability to adapt to a broad range of host-associated and treatment-associated stresses. However, the mechanisms underlying how Mtb dynamically regulates its growth and physiology in response to stress remain incompletely understood. Understanding the gene regulatory activities of transcription factors (TFs) under different environmental or stress conditions could help inform interventions that modulate Mtb growth and survival to cure tuberculosis disease.

Several groups have previously characterized Mtb's transcriptional regulatory network (TRN) using experimental and computational approaches (2–9). These efforts have largely relied on two strategies: (1) detailed profiling of the molecular effects of individual TFs on Mtb physiology using recombinant TF induction and disruption strains, and (2) statistically informed TRN inference using data from large transcriptomic compendia.

In principle, TRNs can be empirically assembled from measurements of TF-DNA binding and gene expression under conditions with known TF perturbations. This approach would be expected to enable the inference of direct regulatory interactions between TFs and their putative target genes, which would be expected to exhibit altered expression in response to TF perturbations and provide evidence of TF binding events proximal to a gene. To leverage this strategy, we previously engineered a library of Mtb recombinant TF induction (TFI) strains (2, 6). We profiled transcriptomes in 208 TFI strains using DNA microarrays [GSE59086, (6, 10)] and detected ~16,000 ChIP-seq binding events for 154 TFs (~80% of all Mtb TFs) and 2,843 genes (~70% of all Mtb genes) (3, 10). While these ChIP-seq and microarray experiments yielded important insights into the regulatory programs active during Mtb broth culture, they also possessed several limitations. For example, our microarray profiling efforts were unable to measure changes in expression for 1,190 genes (~30% of Mtb genes) (6), and our ChIP-seq profiling efforts were unable to detect TF binding for 1,040 genes (~26% of Mtb genes) (3). Moreover, these data were limited to log-phase growth of the Mtb laboratory strain H37Rv in 7H9 media. These excluded condition-specific interactions relevant to other environments or strains. Thus, significant gaps remained in the ability to comprehensively identify TF-gene regulatory interactions using only experimental approaches alone.

Bioinformatic network inference provides a useful complementary strategy for assembling TRNs. These statistically informed approaches utilize large-scale expression compendia (comprising transcriptomic profiles across diverse biological conditions) to enable the inference of regulatory relationships across a multitude of conditions. However, these computational strategies are constrained by two limitations. First, large and biologically diverse gene expression data are needed to enable the identification of high-confidence statistical associations between TFs and their putative target genes (11). Second, network inference algorithms differ in the assumptions made on the training data and on the interpretation of TF-gene associations. Biologically diverse gene expression data may be curated from public microarray (4, 10) or RNA-seq (7, 12, 13) data. However, the statistical assumptions underlying most network inference methods are often biologically inaccurate.

We previously performed computational network inference analyses and were able to infer only 598 clusters of coregulated gene expression for 3,922 genes (4). Others have performed similar analyses and inferred either 80 clusters for 3,906 genes (7) or 560 co-regulated gene modules for 3,912 genes (5). While these studies successfully uncovered novel regulatory interactions underlying Mtb stress adaptation, none of their network models comprehensively revealed transcriptional programs for each of Mtb's 214 TFs, and none directly estimated TF activities [TFAs: the extent of regulation that each TF exerts on its regulon (14)] under different experimental conditions. Network inference studies for other microbes, such as the DREAM5 challenge for *E. coli* and *S. aureus* (15), demonstrated that robust TRNs may be assembled by integrating the regulatory relationships inferred by different network inference algorithms. We hypothesized that applying a similar “wisdom of crowds” approach to aggregate complementary TRNs from different inference methods would yield a more comprehensive and higher quality Mtb TRN than from any single method alone.

Here, we assembled a biologically diverse and batch-corrected Mtb RNA-seq gene expression compendium. We integrated this RNA-seq compendium with a perturbative TFI microarray dataset to infer a comprehensive Mtb TRN that included 214 TFs and 3,978 genes. We used this TRN to estimate TFA profiles corresponding to individual RNA expression profiles. We trained an interpretable machine learning regression model using growth phenotypes from a pooled TFI screen (16) and calculated TFAs for individual TFI strains. We demonstrated that this regression model can accurately predict Mtb fitness under stress conditions such as hypoxia.

## 2 Methods

### 2.1 TFI microarray expression compendium assembly and normalization

Microarray expression data corresponding to TFI strains were downloaded from GEO (GSE59086). Experimental group numbers were assigned to each sample based on the identity of each strain. The *Rv2160A* gene fully encompasses the *Rv2160c* gene, so the Rv2160A and Rv2160c samples were combined into a single Rv2160 TFI strain group. This resulted in 208 TFI strain groups. These 208 strain groups included Rv0560, Rv3164c, and Rv3692, which were initially considered hypothetical during in TFI strain construction (6) but later determined to not be true Mtb TFs (10). However, for the purpose of the analyses presented here, each of these 208 strains will be referred to as TFs. Smooth quantile normalization (17) was performed using *PySNAIL* (18) using the assigned group definitions (Supplementary Table 1).

### 2.2 RNA-seq expression compendium assembly, quality control, and normalization

The NCBI Sequence Read Archive (SRA) was queried with “*Mycobacterium tuberculosis*” for RNA expression samples containing raw FASTQ sequencing reads. Three thousand and ninety eightFASTQ sequencing reads were downloaded and combined with FASTQ sequencing reads from 312 unpublished RNA-seq profiles generated by our labs. We aligned these sequencing reads against the NC_000962.3 Mtb H37Rv reference genome using Bowtie 2 (19). Read counts were compiled using *featureCounts* (20). Samples with fewer than 400,000 total gene counts and duplicated samples were excluded from further analysis. Sequencing counts were normalized by transcripts per kilobase million (TPM). Group definitions representing unique experimental conditions were assigned to each sample; biological replicates were given the same group definitions. Smooth quantile normalization (17) was performed using *PySNAIL* (18) using the assigned group definitions (Supplementary Table 2). Quality data, adapter and quality trimming statistics, and alignment and counts metrics were compiled and assessed using *MultiQC* (21).

### 2.3 UMAP visualization and cluster estimation

RNA expression compendia and TFAs were visualized by Uniform Manifold Approximation & Projection (UMAP) (22). Clusters were estimated by *DBSCAN* using Euclidean distance with a minimum cluster size of 3 (23). The ε hyperparameter was optimized for each dataset by varying ε across 50 logarithmically distributed values from 0.1 to 10 and selecting the value of the elbow of the ε vs. Number of Outliers plot. This selection delivers the minimum number of clusters that maximizes inclusion of samples without overfitting the data (Supplementary Figure 1). UMAP and DBSCAN analyses were performed in Python using their implementations in *umap-learn* and *scikit-learn* (24).

### 2.4 Regulatory network inference

We implemented an ensemble of network inference methods based on a selection of methods featured in the DREAM5 challenge (15), based on diversity in underlying statistical approach, predictive performance reported in the DREAM5 study, and the availability of a working implementation. Our initial selection consisted of ARACNe (25, 26), CLR (27), and GENIE3 (28). We chose an ARACNe implementation that employs adaptive partitioning for more efficient processing (25, 26). We used an R implementation of CLR available on CRAN from the *parmigene* package (29). We used an R implementation of GENIE3 available on BioConductor (30). To supplement these methods, we incorporated two other recent network inference approaches: cMonkey2 (31, 32) and iModulon (33). We used a docker image containing a Python implementation of cMonkey2, available at https://hub.docker.com/r/weiju/cmonkey2. For iModulon, our desired output was different from the output of this algorithm implemented by the original authors; we created a custom iModulon implementation in Python based on Sastry et al. (34). We implemented an Elastic Net regularization-based network inference approach in Python using *scikit-learn* (24). Elastic Net is a regularization method that takes advantage of the unique properties of both lasso and ridge regression (35) and performs better than either lasso or ridge regression when predictors are correlated and/or under-determined (36). We modeled each gene individually on the expression of all the TFs, and used the resulting coefficients to both select significant relationships and score those relationships. Descriptions of each inference method and the hyperparameters used are provided in Supplementary Table 3.

Each method was wrapped to produce a ranked list of putative TF regulator-target gene relationships in the order of the inferred strength of the regulatory relationship, from strongest to weakest. Execution was completed using docker images (https://hub.docker.com/repositories/malabcgidr?search=network-inference). Auto-regulatory (self-targeting) relationships were excluded. Hyperparameters were chosen to match either the original publications or the DREAM5 challenge when possible. Execution for each method and optimization of their corresponding hyperparameters were validated by testing against the evaluation scripts provided in the Supplementary material of Marbach et al. (15) and Reiss et al. (32).

A network was generated for each dataset (RNA-seq or TFI microarray) using the 6 inference methods, yielding 12 total constituent networks. Pairwise comparisons between inferred networks were made using rank-biased overlap (RBO) as previously described (37). The base RBO score was calculated with a *p*-value chosen to yield 50% weight for the first 1,000 relationships (*p* = 0.9997325) and a depth capped at about 25,000. RBO was performed using a custom Python implementation borrowing heavily from https://github.com/dlukes/rbo.

### 2.5 Inferred network truncation and aggregation

The 12 constituent networks were combined using Robust Rank Aggregation (RRA) (38). A *p*-value cutoff was calculated from a Monte Carlo simulation of possible Mtb regulatory network sizes. To compute these network sizes, a range of network out-degree distributions was generated, each conforming to 214 regulators, ~4,000 genes, a power-law exponent from −0.5 to −2, and a power-law multiplier ranging from 10^−10^ to 10^10^ on a logarithmic scale. Bounds for the power-law exponent were estimated based on RegulonDB *E. coli* networks (39) and Bhan et al. (40). For each putative out-degree distribution, the size of the network was calculated, yielding a sample of 175,000 plausible sizes. In the ranked list of edges output by RRA, all edges were kept for which the associated *p*-value score was less than the empirical probability of a simulated network having a size greater than or equal to the edge's associated network size (i.e., its rank); all remaining edges were discarded (Supplementary Figure 2).

### 2.6 Principal component analysis

Principal component analysis (PCA) was performed on the inferred networks (after truncating each to the size of the overall aggregate), the dataset-level aggregate networks, and the overall aggregate network, using the 30,912-dimensional space represented by the ranks of edges shared across at least 3 of the truncated inferred networks. All missing edges in each network were assigned a rank of 30,912, the size of the space.

### 2.7 Directionality of TF-gene regulatory interactions

Directionality for TF-gene regulatory interactions was determined using the regression models and measured TFI gene expression values (Supplementary Table 4). Two Elastic Net models and two unpenalized linear models were used to infer direction of regulation based on the sign of the regression coefficients, one of each for each dataset (RNA-seq compendium and TFI microarray profile). We supplemented these regression associations with the directionality of significant differential gene expression (i.e., upregulated vs. downregulated expression) measured from the TFI microarray dataset. Linear models were fit in Python with the *statsmodels* package. Coefficients with an FDR < 0.05 were selected as evidence. Elastic Net models with an R^2^ of < 0.8 were excluded; coefficients that were included by the remaining models were selected as evidence. TFI differential expression from the microarray dataset was filtered using an FDR < 0.05 and requiring at least 2-fold change in either direction. Elastic Net models and TFI differential expression were considered strong evidence, whereas the unpenalized linear models were considered weak evidence. A flow chart depicting how the information from these models and differential expression analyses were used to define up vs. down regulation is shown in Supplementary Figure 3.

### 2.8 TRN validation

TRNs were validated by testing against a literature-curated TRN formed via the union of the H37Rv regulatory networks from BioCyc (41) and Sanz et al. (8). Sanz et al. Supplementary Material S1 was filtered for relationships whose supporting evidence included at least one high-confidence physical methodology: LacZ-promoter fusion, GFP-promoter fusion, proteomic studies, electrophoretic mobility shift assays (EMSA), one hybrid reporter system, and chip-on-chip. This yielded a set of 433 high-confidence regulator-target relationships, including 51 regulators and 160 total target genes, that had little to no dependence on the transcriptional information used to build the constituent networks. The BioCyc regulatory network consisted of 1,565 relationships of 102 regulators on 802 unique targets. The union of these two regulatory networks was taken and used to calculate the Matthews correlation coefficient (MCC), as described previously (42, 43), for each network, truncated to the size of the aggregate network to produce comparable results.

### 2.9 TRN gene ontologies

Gene ontology enrichment analysis was performed to characterize biological functions for genes associated with each TF (44, 45). For each TF, genes identified as upregulated, downregulated, or regulated in both directions were analyzed for GO enrichment at an FDR < 0.05. All identified GO annotations that had a child annotation also identified for a given TF were removed for simplicity. Results were filtered to TFs receiving at least 3 remaining significant GO enrichments for further manual inspection and analysis (Supplementary Table 5). TFs with an annotated name and considered to have a literature-supported role listed in the Mycobrowser annotation (46) were assessed for network validation (Table 1). GO analysis was performed in Python using the *goatools* package (47). Gene ontology data was taken from the 2024-06-17 release of go-basic.obo from the Gene Ontology knowledgebase (48) (https://purl.obolibrary.org/obo/go/releases/2024-06-17/go-basic.obo), and mappings to Mtb genes were taken from the European Bioinformatics Institute GOA project, release 20240805 (https://ftp.ebi.ac.uk/pub/databases/GO/goa/proteomes/30.M_tuberculosis_ATCC_25618.goa).

**Table 1:** Network regulators: annotation vs. gene set enrichment analysis of inferred regulon.

**Regulator**	**Name**	**Mycobrowser gene product and function information**	**Inferred Regulon GO Annots. (FDR**<**0.05)**	
			**#**	**Summary**
Rv0353	hspR	Probable MerR family heat shock protein transcriptional repressor. Involved in repression of heat shock proteins. Binds to three inverted repeats in the promoter region of the DnaK operon. Induced by heat shock.	15	Heat and stress response, protein refolding
Rv1657	argR	Probable arginine repressor (AHRC). Regulates arginine biosynthesis genes.	5	Cobalamin, nucleobase, arginine synthesis
Rv2215	dlaT	Dihydrolipoamide acyltransferase, component of pyruvate dehydrogenase. Involved in TCA cycle; converts pyruvate to acetyl-CoA and CO_2_. Also involved in defense against oxidative stress.	45	TCA cycle, respiration, gluconeogenesis, ROS response
Rv2359	zur	Probable zinc uptake regulation protein. Acts as a global negative controlling element, with Zn^2+^ binds operator of repressed genes.	6	Starvation response, ETC; translation repression
Rv2374c	hrcA	Probable heat shock protein transcriptional repressor. Involved in repression of class I heat shock proteins. Prevents heat-shock induction of these operons.	12	Primary metabolism, translation termination
Rv2610c	pimA	Alpha-mannosyltransferase. Involved in the first mannosylation step in phosphatidylinositol mannoside biosynthesis (transfer of mannose residues onto PI).	42	Amino acid synthesis, respiration, cell wall formation
Rv2720	lexA	Repressor. Represses genes involved in nucleotide excision repair and SOS response. Binds 14-bp palindromic sequence.	10	DNA repair
Rv3301c	phoY1	Probable transcriptional regulatory protein PhoU-homolog 1. Involved in regulation of active transport of inorganic phosphate across the membrane.	6	Respiration
Rv3417c	groEL1	60 kDa chaperonin 1 (protein CPN60-1). Prevents misfolding, promotes refolding and proper assembly of unfolded polypeptides generated under stress conditions.	15	Heat and ROS response, protein refolding, Arg synthesis
Rv3574	kstR	Transcriptional regulatory protein (probably TetR-family). Involved in transcriptional mechanism. Predicted to control regulon involved in lipid metabolism.	10	Cholesterol metabolism, lipid synthesis
Rv0491	regX3	Possible antitoxin.	3	RNA processing
Rv0599c	vapB27	Possible antitoxin.	6	Growth regulation, toxin sequestration
Rv0608	vapB28	Possible antitoxin.	6	Growth regulation, toxin sequestration
Rv0623	vapB30	Possible antitoxin.	9	Growth regulation, toxin sequestration, RNase activity
Rv1560	vapB11	Possible antitoxin.	12	Growth regulation, nuclease activity
Rv2009	vapB15	Antitoxin.	9	Growth regulation, RNase activity, gene expression regulation
Rv2760c	vapB42	Possible antitoxin.	5	DNA repair

### 2.10 Calculating transcription factor activity profiles from network component analysis

TFAs for each expression profile were computed using Robust Network Component Analysis (ROBNCA) (49). ROBNCA was implemented in Python, using code adapted from https://github.com/CovertLab/WholeCellEcoliRelease/tree/00cf7738cb/reconstruction/ecoli/scripts/nca (50).

### 2.11 Associating network activity with bacterial fitness

We constructed a model associating mycobacterial growth with TFAs estimated from gene expression data. The GSE59086 microarray dataset was again used as a broad measure of TFI conditions, with relative growth data for 194 matching TFI conditions added from Ma et al. Supplementary Table S1 as training data (16). TFAs were computed from log_2_ fold-change expression using the control strengths calculated by ROBNCA from the aggregate network and RNA-seq compendium. An Elastic Net model was trained to regress growth on TFAs, using a grid search cross-validation scheme to optimize hyperparameters. The model was implemented in Python using the *scikit-learn* package (24).

### 2.12 Hypoxia time-course experiment

Wildtype H37Rv (ATCC 27294) transformed with a control anhydrotetracycline (ATc)-inducible expression vector (H37Rv::pEXCF-empty, which does not induce recombinant gene expression) were cultured under in Middlebrook 7H9 with the oleic acid, bovine albumin, dextrose, and catalase (OADC) supplement (Difco) and with 0.05% Tween 80 at 37°C. H37Rv::pEXCF-empty was grown with the addition of 50 μg/mL hygromycin B to maintain the plasmid and induced with 100 ng/mL ATc 1 day prior to onset of hypoxia. For hypoxia, strains were cultured in oxygen-limited conditions (1% aerobic O_2_ tension) for 7 days, followed by reaeration on day 7–12, initiated by transferring cultures into continuously rolled bottles with 5:1 head space ratio using methods described previously (2, 51–53). Bacterial survival and growth were enumerated by plating for colony forming units (CFU) on Middlebrook 7H10 solid media plates using standard microbiological methods.

Transcriptomes were generated by RNA-seq from bacterial cultures sampled from the aforementioned conditions using methods described previously (54). Briefly, bacterial pellets suspended in TRIzol were transferred to a tube containing Lysing Matrix B (QBiogene) and vigorously shaken in a homogenizer. The mixture was centrifuged, and RNA was extracted from the supernatant with chloroform, followed by RNA precipitation by isopropanol and high-salt solution (0.8 M Na citrate, 1.2 M NaCl). Total RNA was purified using a RNeasy kit following the manufacturer's recommendations (Qiagen). rRNA was depleted from samples using the RiboZero rRNA removal (bacteria) magnetic kit (Illumina Inc., San Diego, CA). Illumina sequencing libraries were prepared from the resulting samples using the NEBNext Ultra RNA Library Prep kit for Illumina (New England Biolabs, Ipswich, MA) according to the manufacturer's instructions, and using the AMPure XP reagent (Agencourt Bioscience Corporation, Beverly, MA) for size selection and cleanup of adaptor-ligated DNA. We used the NEBNext Multiplex Oligos for Illumina (Dual Index Primers Set 1) to barcode the libraries to enable sample multiplexing per sequencing run. The prepared libraries were quantified using the Kapa quantitative PCR (qPCR) quantification kit and sequenced at the University of Washington Northwest Genomics Center with the Illumina NextSeq 500 High Output v2 kit (Illumina Inc., San Diego, CA). The sequencing run generated an average of 75 million base-pair paired-end raw read counts per library. Read alignment and gene expression estimation was carried out using a custom processing pipeline in R that harnesses the Bowtie 2 utilities (19, 55), which is publicly accessible at https://github.com/robertdouglasmorrison/DuffyTools and https://github.com/robertdouglasmorrison/DuffyNGS.

Corresponding TFAs were estimated by applying the ROBNCA-parameterized TRN to each gene expression profile. These were applied as inputs to the TFA–fitness Elastic Net model to predict relative fitness level at each time point. Predictions were faceted by day and biological replicate. TF expression, activity, and impact (product of respective regression coefficient and TFA) were normalized to z-score across all samples and TFs for comparison. These were then grouped by condition (hypoxic or normoxic), and TFs with mean impact z-score of a greater absolute value than 1.96 were selected for further analysis and visualization.

### 2.13 False discovery rate correction

False discovery rate correction was performed using the two-stage Benjamini-Krieger-Yekutieli method (56).

## 3 Results

### 3.1 Assembly of a large and biologically diverse Mtb gene expression compendium

**Figure 1:**
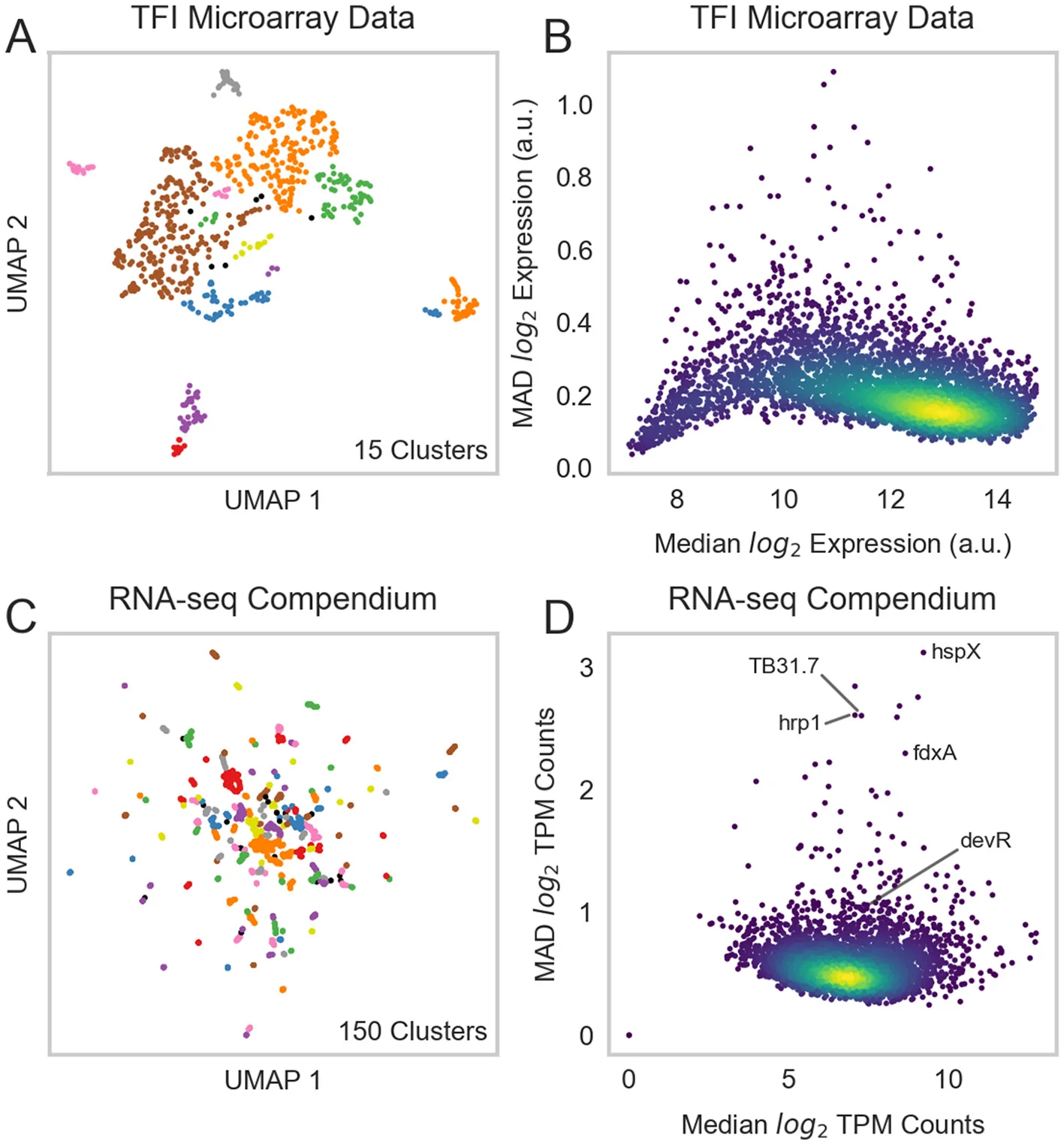
A biologically diverse Mtb RNA expression compendium. **(A)** UMAP visualization of biological diversity in the TFI microarray data. TFI data were batch-corrected by smooth quantile normalization before computing the UMAP. Density-based spatial clustering (DBSCAN) was performed on the UMAP to identify clusters of samples with similar gene expression. UMAP and DBSCAN analyses revealed 15 total gene expression clusters in the TFI dataset. **(B)** Median vs. median absolute deviation (MAD) plot of expression for each gene across the TFI dataset. Each point represents a gene. Median expression and MAD were calculated for each gene across the 698 samples. Colors reveal point density (yellow: high density, blue: low density). **(C)** UMAP visualization of samples from the normalized and batch-corrected RNA-seq compendium determined by gene expression. UMAP and DBSCAN analyses reveal 150 clusters of samples with similar gene expression. **(D)** Median vs. MAD plot of expression for each gene across the RNA-seq compendium.

Our previous efforts to characterize Mtb's TRN relied on microarray expression profiles from recombinant TFI strains as perturbative training data [GSE59086, (6)]. However, while these data enabled some detailed characterization of Mtb's transcriptional programming during log-phase broth culture, they lacked biological diversity. UMAP and DBSCAN analyses reveal that expression profiles from these 698 microarray experiments and 208 TFI conditions formed only 15 unique expression clusters (Figure 1A). This poor biological diversity stems from the original experimental design, in which each TFI strain was grown to log-phase in albumin-dextrose-catalase (ADC)-supplemented 7H9 media before RNA isolation. In addition, microarray technologies possess poor sensitivity and limited dynamic range (57). We found that 101 genes in this dataset did not possess expression measurements >10 log_2_ units, indicating poor sensitivity (Figure 1B, Supplementary Table 1). Moreover, the median absolute deviation (MAD) was small (< 1) for nearly all genes, indicating limited dynamic range. These limitations motivated the need to assemble a larger and more biologically diverse RNA expression compendium.

Thus, we curated an RNA-seq expression compendium using samples from the NCBI Sequence Read Archive (SRA) and unpublished samples from our own labs. We aligned, filtered, normalized, and batch-corrected these samples to form our final compendium (see Section 2). Batch correction is an important pre-processing step for unifying data from different sources that has been frequently overlooked in previous Mtb RNA expression compendium analyses (4, 7, 12, 13). After performing these pre-processing steps, our final compendium comprised 3,410 RNA-seq samples from 1,422 experimental conditions (Supplementary Table 2). Expression counts for the RNA-seq compendium can be queried at https://tfnetwork.streamlit.app/.

UMAP and DBSCAN analyses validated the biological diversity of our batch-corrected RNA-seq expression compendium (Figure 1C). These analyses identified 150 unique expression clusters. The dynamic range and variation in gene expression was significantly greater in this RNA-seq expression compendium than in the TFI microarray dataset (Figure 1D). Of note, most genes with high variation (high MAD) were well-characterized stress response genes [e.g., Rv2031c *(hspX)*, Rv2626c *(hrp1)*, Rv2623 *(TB31.7)*, and Rv2007c *(fdxA)*]. Interestingly, many of these genes possessed higher variation than the commonly studied stress response regulator Rv3133c *(devR)*. These overall results are consistent with expectation, as most stress response genes would be expected to only be induced in the presence of their specific stressor.

### 3.2 Transcriptional regulatory network interactions enrich for shared function

**Figure 2:**
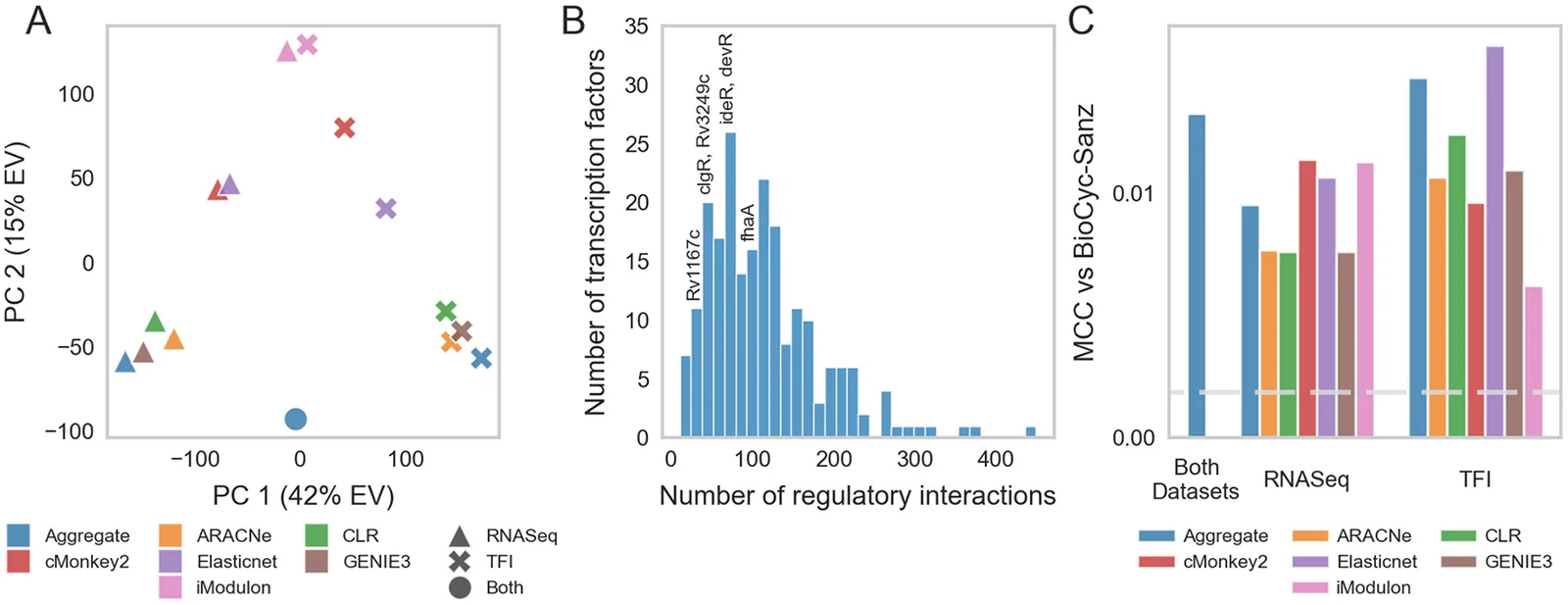
Mtb transcriptional regulatory network. **(A)** PCA was performed on individual and aggregate TRNs generated by different network inference algorithms. PC1 is highly correlated with the dataset used; PC2 is highly correlated with the method used. **(B)** Out-degree distribution of TF-gene interactions (edges) from the overall aggregate network. The distribution of transcriptional regulatory programs largely follows the power law. Highly impactful regulators of hypoxia, in addition to the general stress response regulator Rv3133c (*devR*) are annotated. **(C)** Validation of each network against literature-curated TRNs. The Matthews correlation coefficient (MCC) was computed between each individual or aggregate TRN and the union of manually curated TRNs by Sanz et al. (8) and BioCyc (41). Blue bars depict the MCC for aggregate networks; the other colors depict the MCC for the individual inferred networks. The horizontal dashed line represents the 95th percentile MCC performance of 1,000 randomly generated networks.

Network inference studies in other bacteria have shown that aggregate TRNs, formed by integrating regulatory relationships derived from multiple inference algorithms, outperform networks generated by individual methods (15). To comprehensively model Mtb transcriptional regulation, we employed a “wisdom of crowds” ensemble inference approach. Using our RNA-seq compendium, we generated a set of 6 TRNs using different network inference methods (see Section 2). These methods were selected because they have either been shown to be sensitive to distinct types of regulatory relationships (15) or have been previously applied to infer regulatory relationships in Mtb (4, 5, 7). To further diversify the inferred networks, we also applied these methods to the TFI microarray dataset. Collectively, these activities yielded 12 networks that described ~783,400 unique relationships between 214 regulators and 4,029 target genes. Pairwise comparisons revealed that the networks predicted by the different inference methods were mostly dissimilar (Supplementary Figure 4). From these, we constructed aggregate networks for the RNA-seq compendium, TFI microarray dataset, and the combination of both datasets using Robust Rank Aggregation (RRA) (38) (see Section 2). Principal component analysis on the individual and aggregate networks corroborated the substantial diversity derived from the different network inference methods and datasets (Figure 2A).

The final overall aggregate network model comprised 24,543 regulatory relationships linking 214 transcriptional regulators with 3,978 target genes. Among these relationships, 16,292 were associated with transcriptional activation, 3,247 with transcriptional repression, 1,093 with context-dependent regulation, and 3,911 with undetermined directionality (Supplementary Table 4). These relationships represent both direct biophysical interactions as well as indirect regulatory relationships mediated through intermediate regulators. The distribution of regulatory relationships for each TF largely followed a power law distribution; this is consistent with the scale-free network architectures found in the transcriptional networks of other bacteria (Figure 2B, Supplementary Figure 2C) (58). The networks are accessible at https://tfnetwork.streamlit.app/. TF-gene relationships are provided in Supplementary Table 4.

To validate our aggregate network, we benchmarked it against previously established regulatory relationships gleaned from the literature (see Section 2). We evaluated the consensus between this high-confidence regulatory interaction dataset and our inferred regulatory networks using the Matthews correlation coefficient (MCC) (42, 43). We found that all of the inferred networks possessed significant MCCs and that the overall aggregate network outperformed most of the networks derived using only one network inference method (Figure 2C).

We also assessed the extent to which the regulatory relationships captured by the aggregate network preserved biologically meaningful functional relationships between TFs and target genes. For well-studied TFs, gene ontologies for their predicted target genes were highly consistent with what has been reported for that TF in the literature (Table 1, Supplementary Table 5A). For example, Rv3574 (*kstR*) is a TF that has been linked to cholesterol metabolism (59) and the target genes associated with *kstR* possess gene ontology annotations linked to cholesterol metabolism (Table 1). Additionally, toxin-antitoxin target genes were enriched for growth regulation, highlighting that the regulatory relationships captured by the aggregate network include indirect regulatory interactions. These results suggest that the ontologies and functional annotations predicted for poorly characterized TFs may provide experimentally testable insights on their function. This is one of the major advances from the aggregate network.

### 3.3 Network component analyses reveal condition-specific TF activities

Understanding when TFs actively regulate their target genes can reveal mechanistic insights into bacterial physiology and stress response. Network component analysis (NCA) is an efficient way of estimating TFA profiles from expression data by using a TRN to perform matrix decomposition (14). Robust NCA (ROBNCA) is a variant of NCA that improves the performance of NCA calculations on noisy data with outlier measurements (49). We applied ROBNCA to estimate TRN control strengths and TFAs corresponding to each sample in our TFI microarray and RNA-seq compendium.

**Figure 3:**
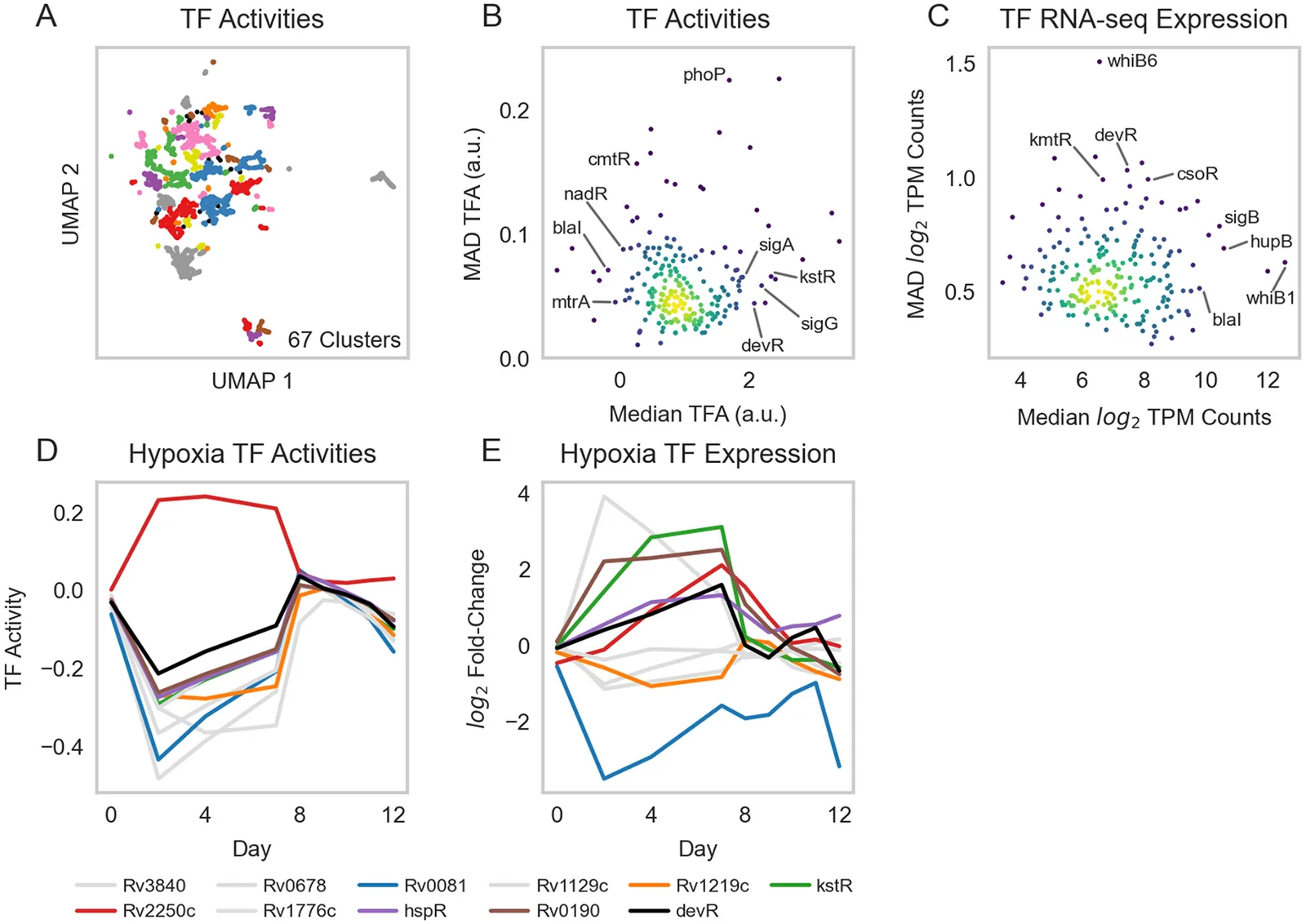
Transcription factor activities. **(A)** UMAP visualization of samples from the normalized and batch corrected RNA-seq compendium as determined by TFA. UMAP and DBSCAN analyses reveal 67 clusters of samples with similar TFAs. **(B)** Median vs. MAD plot of activity for each TF across the RNA-seq compendium. **(C)** Median vs. MAD plot of expression for each TF across the RNA-seq compendium. **(D)** TFAs highly active under hypoxia, as calculated using control strengths derived from ROBNCA calculation on the RNA-seq compendium, throughout a 12-day hypoxia-reaeration experiment. TFs plotted in color have been previously found to correlate with hypoxia (7, 60–62). **(E)** Corresponding expression levels of TFs with high activity during hypoxia. Rv3133c (*devR*) is included as a point of reference.

We applied ROBNCA to our RNA-seq compendium using the aggregate TRN inferred from the RNA-seq compendium and TFI microarray data (Supplementary Table 6). UMAP and DBSCAN analyses revealed less biological diversity in ROBNCA-predicted TFAs than RNA expression profiles alone (67 clusters of TFAs vs. 150 clusters of expression; Figure 3A). Amongst the TFs with the highest level of median activity were the lipid metabolism regulatory Rv3574 (*kstR*) and sigma factor Rv0182c (*sigG*) (Figure 3B). Consistent with expectation, the well-characterized stress response regulators Rv0757 (*phoP*) and Rv1994c (*cmtR*) were amongst the TFs with the highest TFA MAD.

Interestingly, the distribution of TFAs appeared different from that of TF expression levels measured for each RNA-seq sample across the compendium (Figure 3C). We tested the correlation of expression level vs. activity for each TF across the entire compendium and found that expression and activity were poorly correlated across the dataset (Pearson's *r* = 0.22 ± 0.23 median ± MAD). Six TFs were strongly correlated (| Pearson's *r* | ≥ 0.7), 25 TFs were moderately correlated (0.7 > | *r* | ≥ 0.5), and 60 TFs were weakly correlated (0.5 > | *r* | ≥ 0.3). These suggest that TF expression is not the key determinant for TFAs for most TFs. Rather, expression and activity convey two distinct but complementary insights into transcriptional regulation, highlighting the importance of accounting for network interactions when investigating transcriptional regulation.

To evaluate the performance of our TRN in predicting TFAs under stress, we performed experiments that profiled Mtb expression during hypoxia and reaeration. We grew the Mtb H37Rv empty vector control strain from our TFI strain library (2, 6) to exponential phase in 7H9 media supplemented with OADC and subjected cells to 7 days hypoxia, followed by 5 days reaeration (see Section 2). We sampled and sequenced RNA at several time points to profile changes in Mtb expression under hypoxia-reaeration stress. We estimated TFAs corresponding to each time point using our ROBNCA-trained TRN and evaluated the TF with the greatest predicted changes in activity during hypoxia (Figure 3D). Our analyses predicted significant changes in activity for several TFs that were previously described in the literature, including the general stress response regulator Rv3133c (*devR*), Rv0081 (early responder under hypoxia), and Rv2250c (late responder under hypoxia) (7, 60–62). Interestingly, changes in TFA during hypoxia and reaeration were not strongly correlated with changes in expression for these TFs (Figure 3E), further supporting our interpretation that TFA and expression convey different biological insights.

### 3.4 Transcription factor activity profiles can predict bacterial fitness under stress

Because transcriptional programs mediate Mtb's response to changing environmental conditions, we asked whether our TRN could predict the Mtb growth fitness under stress. To test this hypothesis, we applied Elastic Net regularization to construct an interpretable machine learning regression model that could predict Mtb fitness from calculated TFA profiles alone. We trained this model using the TFAs computed by ROBNCA for each recombinant TFI strain from the microarray expression profiles and paired with fitness phenotypes that we previously measured in a Transcriptional Regulator Induced Phenotype (TRIP) screen (16). Both of these datasets were measured under log-phase, aerated culture conditions. The resulting TFA–fitness regression model explained ~80% of the observed variation in growth between the TFI strains in the TRIP screen (Supplementary Figure 5).

**Figure 4:**
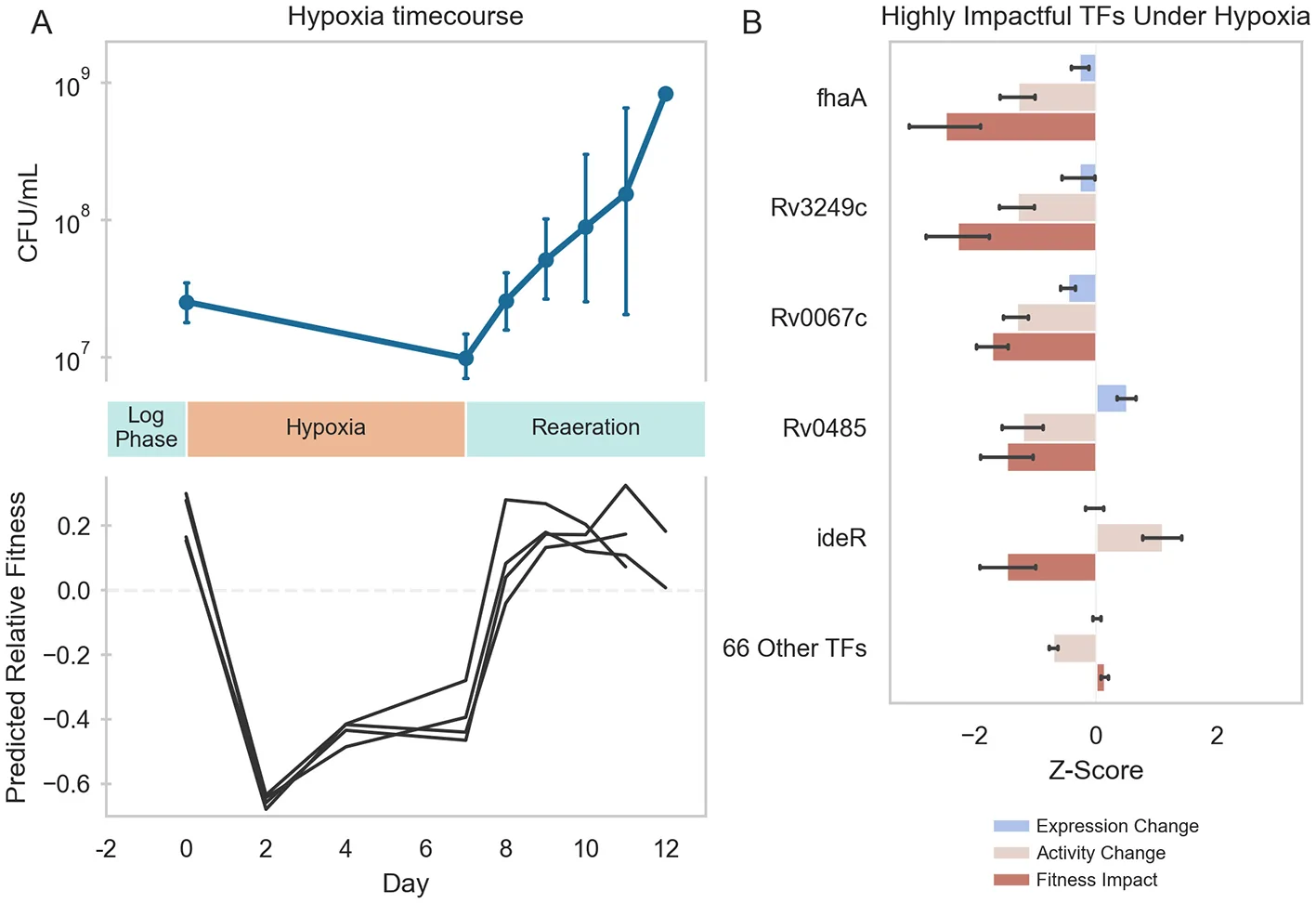
Machine learning predictions of Mtb fitness under hypoxia-reaeration stress. **(A)** *Top*: Mtb growth during hypoxia and reaeration. Mtb H37Rv::pEXCF-empty cells were to log-phase in 7H9 medium supplemented with OADC for 2 days (Day 0). Cells were subjected to 7 days hypoxia, followed by 5 days reaeration. Hypoxia induced growth arrest. Reaeration induced resumption of growth. *Bottom*: Elastic Net model predictions of Mtb fitness during hypoxia and reaeration. TFAs were estimated for each RNA-seq sample collected at different time points during the hypoxia-reaeration experiment. TFAs for each time point were supplied as inputs to the Elastic Net model to predict fitness at each time point. Model predicts decreased fitness during hypoxia and restored fitness during reaeration. Model predictions were made for each biological replicate in the experiment (depicted as separate lines). **(B)** The Elastic Net-predicted transcriptional regulators of Mtb fitness during hypoxia. Fitness impact scores were computed for each TF using the Elastic Net model. The fitness impact score for each TF is computed from its TFA and the regression coefficient for that TF in the Elastic Net model. Fitness impact scores were averaged over the period of hypoxia (days 2–7). Five TFs were predicted to be highly influential for growth arrest during hypoxia. Depicted is the gene expression, ROBNCA-predicted activity, and Elastic Net-predicted fitness impact for each TF. Error bars represent 95% confidence intervals.

To determine if this TFA–fitness regression model could predict changes in Mtb fitness in conditions not included in the training data, we predicted the fitness of wildtype H37Rv cells undergoing hypoxia and reaeration stress based on time-varying RNA expression data during stress each phase. From the TFA profiles calculated for hypoxia-exposed cells, the TFA–fitness regression model predicted a significant decrease in growth over the entire hypoxia period (Figure 4A). From the TFA profiles calculated for cells under reaeration, the model predicted a recovery in Mtb growth comparable to log-phase culture. The predicted kinetics of shifts in growth aligned with the experimental measurements of Mtb bacteriostasis during hypoxia and regrowth during reaeration, thus validating the model predictions. Importantly, our TFA–fitness regression model performed better than a regression model trained only on TF expression (Supplementary Figure 6). These results further supported our hypothesis that TFAs more effectively capture condition-specific transcriptional regulation than TF expression alone, thus implying that the activation and regulation of transcriptional programs under hypoxia and reaeration involve non-linear mechanisms.

Because the TFA–fitness regression model is directly interpretable, we examined which TFAs most strongly predicted the fitness changes under hypoxia and reaeration. Our TFA–fitness model predicted that growth restriction during hypoxia is primarily driven by 5 TFs whose TFA profiles changed significantly during hypoxia [Rv0020c (*fhaA*), Rv3249c, Rv0067c, Rv0485, and Rv2711 (*ideR*)] (Figure 4B). Again, TF activity was a stronger predictor of fitness impact than expression. Importantly, each of these TFs possesses direct or indirect links to hypoxia in the literature (Supplementary Table 7) (9, 60, 83–85). These results validate the ability of our model to directly predict mechanisms underlying Mtb stress response biology.

## 4 Discussion

Understanding the molecular drivers of phenotypic changes in an organism is a fundamental goal in biological research. In this study, we applied machine learning approaches to construct an interpretable TFA–fitness regression model that could predict changes in Mtb growth under stress. Our model builds upon previous experimental profiling and network inference modeling efforts to characterize Mtb's TRN by integrating the data and algorithms from prior studies (2–7, 14, 15, 49). Moreover, by training on Mtb fitness profiles from TRIP screens, our model can directly predict growth phenotypes from condition-specific gene expression profiles alone.

Our “wisdom of crowds” approach for inferring transcriptional regulatory interactions delivered significant enrichment of known regulatory relationships while also broadening the scope of represented experimental conditions. Our resulting TRN is substantially larger than the networks inferred by many individual algorithms and performed better at recovering experimentally validated interactions (Figure 2C). This highlights the utility of ensemble inference algorithms (15).

Importantly, our results demonstrate how network models can generate experimentally testable hypotheses in at least two ways. First, our gene ontology enrichment analysis revealed significant associations between the annotated function of a TF's target genes and the condition-specific regulatory roles of the TF. It is important to note that the regulatory relationships identified by our aggregate TRN include both direct physical interactions between a TF and its putative target gene as well as indirect associations mediated by other factors. Both direct and indirect regulatory associations are important for coordinating changes in bacterial physiology (63), so it is expected that both types of interactions share annotated ontologies. Because ~25% of Mtb genes lack functional annotation (64), we think that the regulatory relationships identified in our TRN can generate hypotheses for the functions of poorly characterized or unknown genes (Supplementary Table 5).

Second, we demonstrate that TFA regression models can be trained to predict Mtb fitness under stress. Notably, we show that our TFA–fitness regression model was able to predict Mtb growth and bacteriostasis under hypoxia and reaeration—environmental conditions not used for training the TFA regression model. Thus, our results suggest that TFAs are a useful determinant of condition-specific changes in bacterial growth. Moreover, we show that TFAs are more predictive of growth phenotypes than TF expression alone (Supplementary Figure 6). This is consistent with expectation as Mtb uses transcriptional regulation to orchestrate behavioral adaptations to varying environments, including growth phenotypes. Our modeling analyses also reveal which TFAs underlie the predicted bacterial fitness outcomes. This directly generates hypotheses for the mechanisms underlying how TFs and their corresponding transcriptional programs are activated (e.g., via allosteric mechanisms and/or network interactions). Thus, our TRN and TFA–fitness models could potentially inform the identification of regulatory mechanisms mediating Mtb response and adaptation to clinically relevant stress conditions where gene expression profiling data are available. The TFs and target genes highlighted by these models may reveal druggable targets for manipulating Mtb's fitness under stress. In light of the growing crisis of antimicrobial resistance (65) and multi- and extensively-drug-resistant tuberculosis (66), we think our approach will be important for curing tuberculosis disease (67).

More broadly, our work demonstrates how network models can be leveraged for biologically meaningful interpretable machine learning applications. A fundamental challenge in machine learning is the difficulty in understanding how a machine learning model makes predictions (68, 69). We previously demonstrated that machine learning regression models can elucidate metabolic mechanisms underlying antibiotic lethality in *E. coli* (70) as well as predict multidrug interaction outcomes in Mtb (54). Our study here analogously extends this approach by training a regression model on TFAs to predict changes in Mtb growth under stress. The advantage of this strategy over other contemporary machine learning approaches is the explicit utilization of prior knowledge in the form of biological network models, which directly enables the generation of hypotheses for mechanisms linking network interactions to cell phenotypes. These hypotheses can then be experimentally tested (54, 70) and used as the basis for further mechanistic study (71) and investigation of translational potential.

Looking ahead, we envision that our TRN and our TFA–fitness regression model will be useful for several facets of tuberculosis research. We demonstrated that our regression model can predict changes in Mtb fitness under environmental stress from RNA expression profiles alone. Thus, our model may inform on fitness under clinically relevant conditions where standard microbiological tools are unavailable. In addition, there is increasing appreciation that Mtb drug susceptibility is regulated by its environment (72, 73). Our interpretable TFA–fitness regression model can be used to elucidate the molecular mechanisms underlying these phenotypes. Moreover, functional genetic datasets from different technologies are increasingly available (16, 74–79). These data can be applied to train next-generation TFA–fitness regression models with improved predictive power. Finally, detailed characterizations of Mtb clinical strains are now providing significant insights into how mutations and other forms of genomic diversity regulate drug susceptibility in human patients (79–82). We envision the TRN and TFA–fitness regression framework established here can be extended to not only study the mechanisms underlying differences in drug susceptibility amongst clinical isolates but also anticipate drug susceptibility phenotypes of new strains as they are curated.

## Data Availability

The original contributions presented in the study are publicly available. These data can be found here: https://www.ncbi.nlm.nih.gov/geo/query/acc.cgi?acc=GSE292331, https://www.ncbi.nlm.nih.gov/geo/query/acc.cgi?acc=GSE292332, https://www.ncbi.nlm.nih.gov/geo/query/acc.cgi?acc=GSE292408, https://www.ncbi.nlm.nih.gov/geo/query/acc.cgi?acc=GSE292409, https://www.ncbi.nlm.nih.gov/geo/query/acc.cgi?acc=GSE292410, https://www.ncbi.nlm.nih.gov/geo/query/acc.cgi?acc=GSE292636, https://www.ncbi.nlm.nih.gov/bioproject/PRJNA1226619, and https://www.ncbi.nlm.nih.gov/bioproject/PRJNA1226648.
