# Supplementary material for: Predicting fitness in Mycobacterium tuberculosis with transcriptional regulatory network-informed interpretable machine learning
Source: Front Tuberc. Author manuscript; Available in PMC 2025 Jul 17. (PMC12269550; doi:10.3389/ftubr.2025.1500899)
Supplement: Data sheet 1 [file NIHMS2089678-supplement-Data_sheet_1.docx]

Supplementary Material

# Supplementary Figures and Tables

## Supplementary Figures


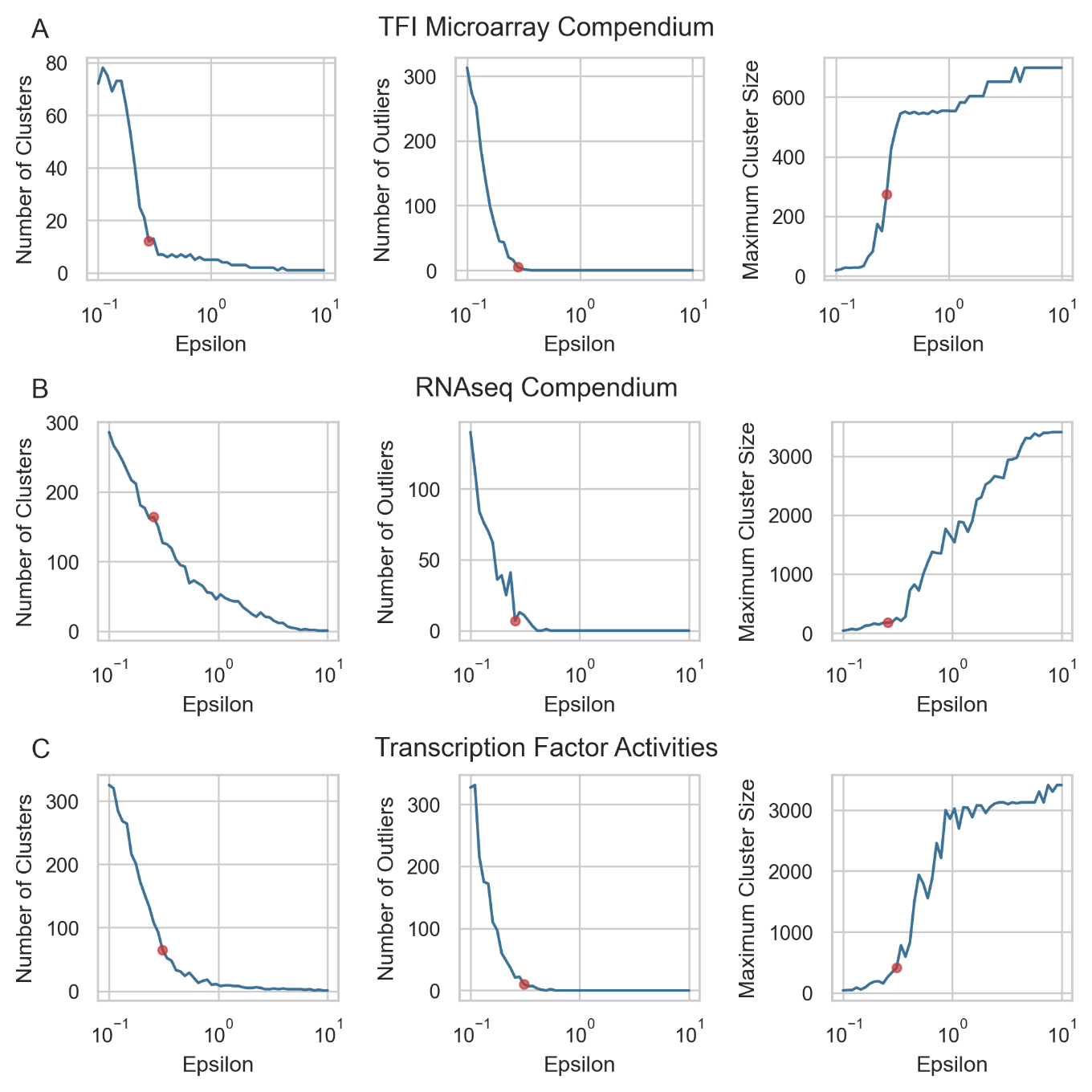


**Supplementary Figure 1 UMAP parameterization.** Hyperparameter optimization was performed on UMAPs from the (A) TFI microarray compendium, (B) RNA-seq compendium, or (C) TFAs calculated from the RNA-seq compendium. ε was varied from 0.1 to 10 on a logarithmic scale and numbers of clusters (left), numbers of outliers (center), and maximum cluster size (right) were computed for each ε. ε was selected from the elbow of the outliers plot (ε = 0.281 for TFI data, 0.256 for RNA-seq compendium, and 0.309 for estimated TFAs).


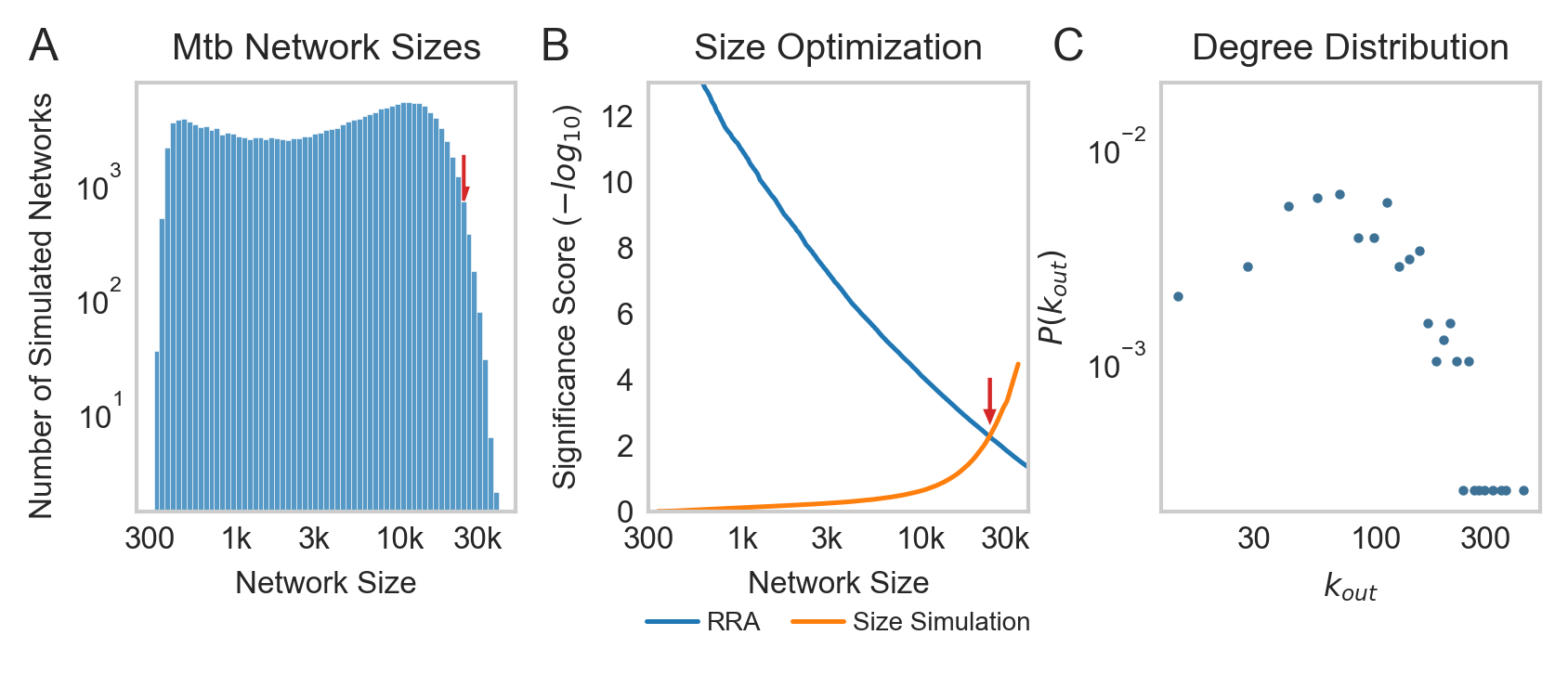


**Supplementary Figure 2 Mtb regulatory network simulation and truncation.** (A) A population of plausible regulatory network sizes following a power-law distribution representative of transcriptional regulatory networks for other organisms (see **Methods**). (B) The ranked edge *p*-values produced by robust rank aggregation (RRA) were compared to the empirical probability of a network taking the given size in the network size simulation. The aggregate network was truncated at the network size that minimizes the RRA *p-*value while satisfying a power-law distribution. (C) The out-degree distribution of this truncated list of edges generally follows the power-law, excepting a few poorly connected regulators.


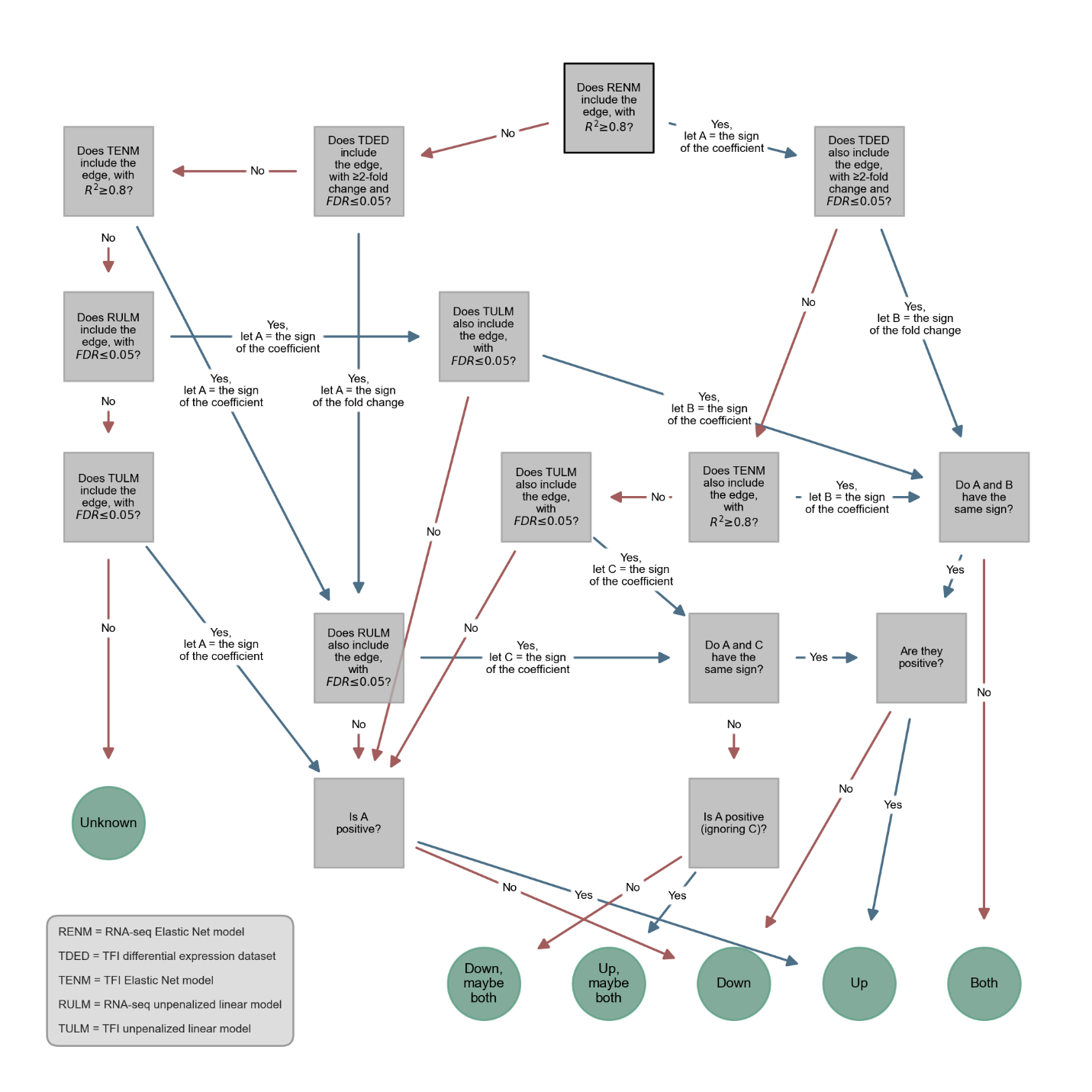


**Supplementary Figure 3 Assignment of activating vs repressing regulatory interactions.** Flow chart depicting the logic used to assign directionality to regulatory relationships. Abbreviations used are defined in the legend in the bottom left.


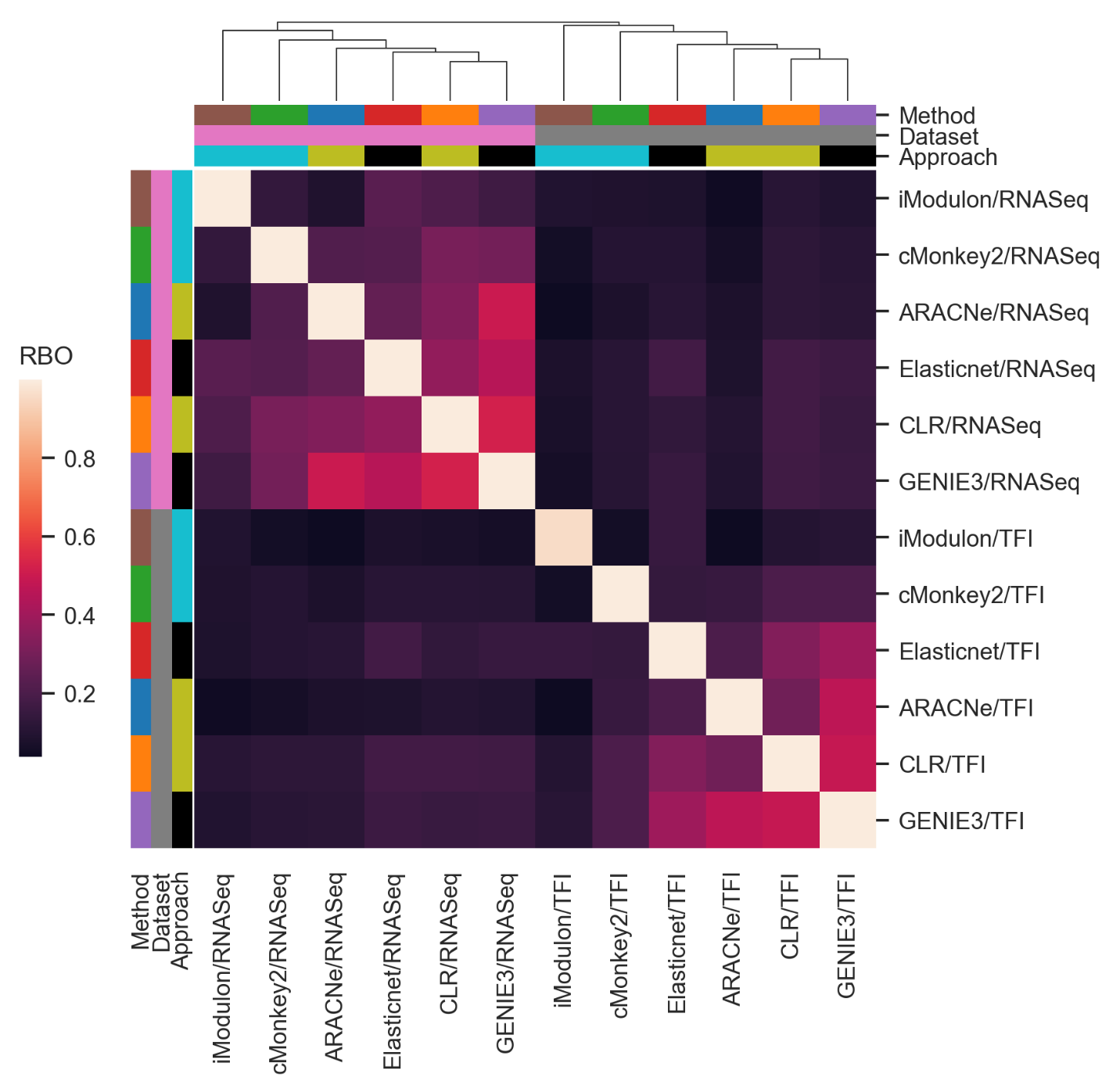


**Supplementary Figure 4 Inferred network similarity.** Heatmap depicting the distance matrix between each inferred network, as measured by rank-biased overlap (RBO), a measure of ranked network similarity (from a minimum of 0, or totally dissimilar, to a theoretical maximum of 1, or identical). Axes have additional color labels corresponding to method (blue=ARACNe, orange=CLR, green=cMonkey2, red=Elasticnet, purple=GENIE3, brown=iModulon), dataset (pink=RNA-seq compendium, grey=TFI screen), and underlying approach (olive=mutual information, cyan=clustering, black=regression).


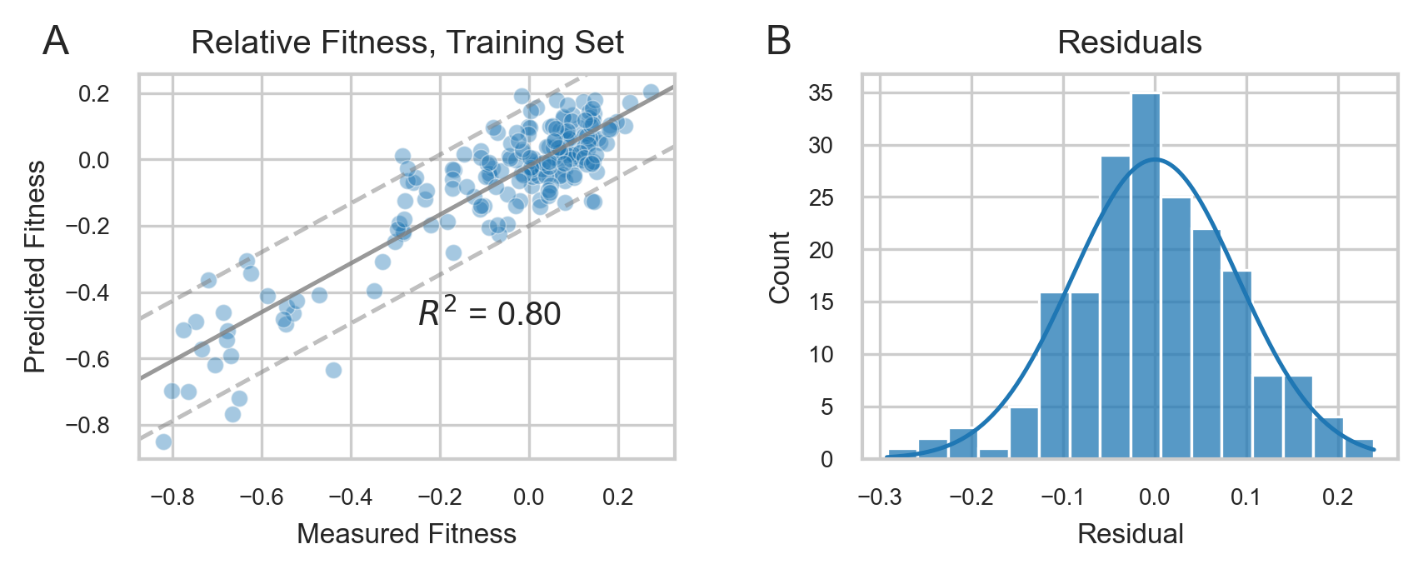


**Supplementary Figure 5** **TFA-fitness regression model performance.** (A) Fitness values predicted by the Elastic Net model versus the experimentally measured fitness values from the TRIP screen. The line of best fit depicts the relationship between predicted and measured values. Dashed lines depict the best fit line plus or minus two standard deviations. (B) Residuals of the model predictions versus measured values form a roughly normal distribution, indicating a lack of bias and overall reliable predictive ability.


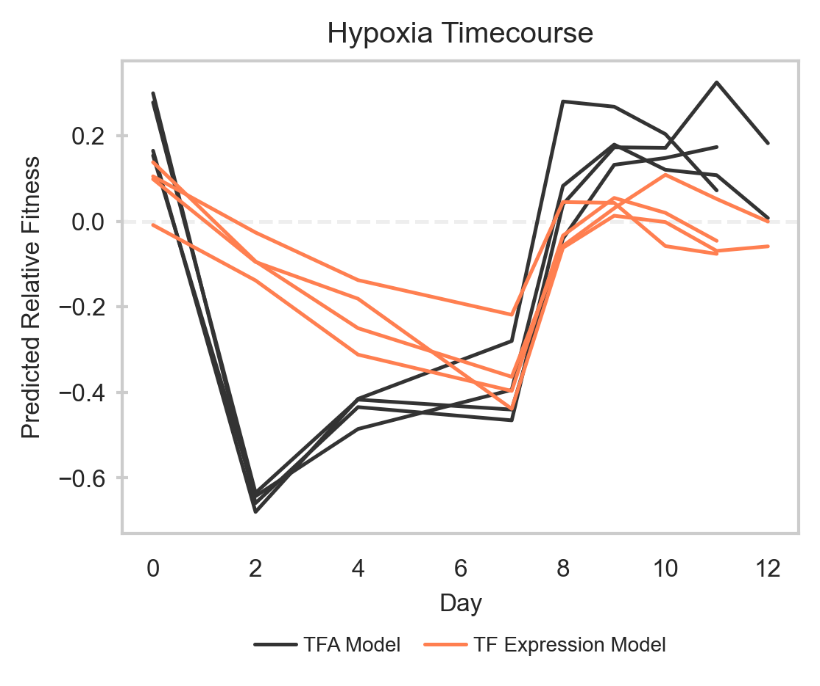


**Supplementary Figure 6** **TF expression hypoxia prediction.** Hypoxia and reaeration fitness changes predicted by an Elastic Net model trained using only TF expression data instead of TFAs, along with the Elastic Net model trained on TFAs. The TFA model shows more dynamic range and more distinct predictions between hypoxic and normoxic timepoints (Welch's *t*-test *p* = 3.8 × 10^-5^ between hypoxia and normoxia in expression model, *p* = 8.3 × 10^-14^ in activity model).

## Supplementary Tables

**Supplementary Table 1 TFI microarray expression data. (A)** Experimental group assignments for each sample in the TFI microarray dataset. **(B)** Smooth quantile normalized microarray expression for all genes and all samples in the TFI microarray dataset. **(C)** Median and MAD expression for each gene. Group assignments were used for batch correction by smooth quantile normalization [18].

**Supplementary Table 2 RNA-seq expression compendium. (A)** Experimental group assignments for each sample in the RNA-seq compendium. Group assignments were used for batch correction by smooth quantile normalization [18]. **(B)** Median and MAD expression for each gene. Full expression data are available on GEO and GitHub (see **Data Availability Statement**).

**Supplementary Table 3 Network inference methods.** Description of transcriptional regulatory network inference methods.

**Supplementary Table 4 Aggregate network directionality of regulation.** Summary of the TF-gene regulatory interactions.

**Supplementary Table 5 TF Gene Ontology assignments.** GO enrichment for each transcriptional program regulated by each TF inferred in the aggregate TRN. (A) Annotated functions and a summary of GO enrichments found for targets from selected TFs with at least 3 significant GO enrichment terms and a non-locus gene name in Mycobrowser [46]. (B) Remaining TFs with at least 3 significant GO enrichments but without an annotated gene name (28 additional TFs). (C) All GO enrichments identified by our analysis were corrected for FDR with a cutoff of 0.05.

**Supplementary Table 6 Transcription factor activities.** Median and MAD expression and activity for each TF in the RNA-seq compendium. Pearson correlation coefficient between TF expression and TFA for each TF across all samples in the RNA-seq compendium.

**Supplementary Table 7 Highly impactful TFAs in hypoxia.** Overview of the top 5 most important TFAs for predicting fitness under hypoxia as identified by the TFA–fitness regression model [9; 60; 83; 84; 85].
